# Elevated De Ritis Ratio as a Predictor for Acute Kidney Injury after Radical Retropubic Prostatectomy

**DOI:** 10.3390/jpm11090836

**Published:** 2021-08-25

**Authors:** Jun-Young Park, Jihion Yu, Jun Hyuk Hong, Bumjin Lim, Youngdo Kim, Jai-Hyun Hwang, Young-Kug Kim

**Affiliations:** 1 Asan Medical Center, Department of Anesthesiology and Pain Medicine, University of Ulsan College of Medicine, Seoul 05505, Korea; parkjy@amc.seoul.kr (J.-Y.P.); yujihion@gmail.com (J.Y.); midorimd@naver.com (Y.K.); jaehyun.hwang.uucm@gmail.com (J.-H.H.); 2 Asan Medical Center, Department of Urology, University of Ulsan College of Medicine, Seoul 05505, Korea; hjhuro@gmail.com (J.H.H.); lbj1986@hanmail.net (B.L.)

**Keywords:** prostate cancer, radical retropubic prostatectomy, De Ritis ratio, acute kidney injury

## Abstract

Acute kidney injury (AKI) is related to mortality and morbidity. The De Ritis ratio, calculated by dividing the aspartate aminotransferase by the alanine aminotransferase, is used as a prognostic indicator. We evaluated risk factors for AKI after radical retropubic prostatectomy (RRP). This retrospective study included patients who performed RRP. Multivariable logistic regression analysis and a receiver operating characteristic (ROC) curve analysis were conducted. Other postoperative outcomes were also evaluated. Among the 1415 patients, 77 (5.4%) had AKI postoperatively. The multivariable logistic regression analysis showed that estimated glomerular filtration rate, albumin level, and the De Ritis ratio at postoperative day 1 were risk factors for AKI. The area under the ROC curve of the De Ritis ratio at postoperative day 1 was 0.801 (cutoff = 1.2). Multivariable-adjusted analysis revealed that the De Ritis ratio at ≥1.2 was significantly related to AKI (odds ratio = 8.637, *p* < 0.001). Postoperative AKI was associated with longer hospitalization duration (11 ± 5 days vs. 10 ± 4 days, *p* = 0.002). These results collectively show that an elevated De Ritis ratio at postoperative day 1 is associated with AKI after RRP in patients with prostate cancer.

## 1. Introduction

Radical prostatectomy is known to be a mainstay of treatment in patients with prostate cancer [1]. Even if robotic or laparoscopic approaches for radical prostatectomy have become increasingly popular, radical retropubic prostatectomy (RRP) is still widely performed, especially in prostate cancer patients with optic neuropathy, increased intracranial pressure, or severe obstructive lung disease [2]. Complications of RRP include bleeding, urinary tract infection, bladder neck contracture, erectile dysfunction, and urinary incontinence [3]. Postoperative acute kidney injury (AKI) is another frequent complication of RRP, with an incidence of 10.4% [4]. Postoperative AKI has been found to be related to progressive chronic kidney disease, prolonged hospitalization duration, higher medical costs, and increased mortality [5]. Therefore, careful perioperative management comprising risk factor evaluations should be performed to reduce the chances of AKI after RRP.

The De Ritis ratio, which is calculated by dividing the aspartate aminotransferase (AST) concentration by the alanine aminotransferase (ALT) concentration [6,7], may predict a prognosis of patients with bladder cancer, renal cell carcinoma, oropharyngeal cancer, pancreatic cancer, cardiac arrest, acute myocardial infarction, or ischemic stroke, and those with SARS-CoV-2 [8,9,10,11,12,13]. However, no studies to date have reported on the association of the De Ritis ratio and AKI after RRP. 

Therefore, this work attempts to identify the independent risk factors, such as the De Ritis ratio, related to postoperative AKI in prostate cancer patients who underwent RRP.

## 2. Materials and Methods

### 2.1. Patients 

This large, single-center, retrospective, observational study analyzed data on patients who had performed RRP at the Asan Medical Center, Seoul, Korea, between January 2009 and November 2020. This study’s design obtained approval from the institutional ethics review board of Asan Medical Center (approval number of 2020-1925). The need for written informed consent was exempted given the retrospective and observational nature of the study’s design. This study adheres to the STROBE guidelines. Exclusion criteria included other malignancies, incomplete medical records, previous kidney transplantations, or known end-stage renal disease. 

### 2.2. Anesthesia and Monitoring 

Routine anesthesia monitoring comprising noninvasive blood pressure, pulse oximetry, electrocardiography, and end-tidal carbon dioxide concentration was performed for all patients. For anesthesia induction, thiopental or propofol was administered. All patients were administered rocuronium bromide to relax the muscles. Maintenance of anesthesia was carried out using a volatile anesthetic (desflurane or sevoflurane) combined with 1.0–4.0 ng/mL remifentanil continuous infusion. Desflurane or sevoflurane was titrated for maintaining a bispectral index between 40 to 60. Plasma solution A (Cheil Jedang Pharmaceutical, Seoul, Korea) was administered at a rate of 4–6 mL/kg/hr. Colloid fluids, including hydroxyethyl starch 6% or albumin 5%, were administered by our institutional protocol and after consideration of the patients’ heart rate, arterial blood pressure, and surgical bleeding. Red blood cells were transfused into patients with intraoperative hemoglobin < 8 g/dL. 

### 2.3. Surgical Protocol 

Vertical midline and extraperitoneal incisions were made from almost below the umbilicus to just above the pubic symphysis. Fat tissue covering the lateral pelvic sidewalls and prostate was dissected to open Retzius’ space. The endopelvic fascia was opened near the pelvic sidewall adjacent to the prostate. The puboprostatic ligaments were incised to open the prostate apex. To reduce bleeding, dorsal vein complex ligation was performed. Pelvic lymph node dissection was proceeded based on clinical risk factors [14]. The anterior urethra was cut sharply to ensure that the length of the striated urethral sphincter remained as long as possible. While pulling the foley catheter toward the bladder, the posterior aspect of the prostate and both pedicles were carefully dissected along with the neurovascular bundles. After the prostate was dissected, the vasa deferentia were cut, and the seminal vesicles were dissected between the remaining bladder neck and prostate. After bladder neck reconstruction, vesicourethral anastomosis was performed.

### 2.4. Data Collection 

All study patients’ medical records were analyzed. The demographics and preoperative laboratory data that were collected for each patient included body mass index, age, American Society of Anesthesiologist (ASA) physical status, history of abdominal surgery, comorbidities (diabetes mellitus, hypertension, coronary artery disease, chronic kidney disease, cerebrovascular accident, or chronic obstructive pulmonary disease), medications (aspirin, clopidogrel, calcium channel blockers, angiotensin-converting enzyme inhibitor or angiotensin II receptor blocker, and beta blocker), Gleason score, tumor stage, and preoperative laboratory values (prostate-specific antigen, hemoglobin, platelet, white blood cell, neutrophil, lymphocyte, platelet/lymphocyte ratio, neutrophil/lymphocyte ratio, estimated glomerular filtration rate [eGFR], uric acid, albumin, AST, ALT, C-reactive protein, and the De Ritis ratio) [15]. The Chronic Kidney Disease Epidemiology Collaboration equation was used to calculate eGFR [16]. 

Intraoperative data included operation duration, crystalloid amount, and 6% hydroxyethyl starch amount. The number of patients who received albumin 5% and red blood cell transfusion and those with extracapsular extension, seminal vesical invasion, positive surgical margins, and pelvic lymph node dissection were recorded. Extracapsular extension was considered to be tumor extension into the periprostatic tissue [17]. Seminal vesical invasion was considered to be the penetration of the muscular coat of the seminal vesicles by the prostate tumor [18]. Positive surgical margins were considered to be the presence of cancer cells at the inked margins [19]. 

Postoperative laboratory data included hemoglobin, platelet, platelet/lymphocyte ratio, neutrophil/lymphocyte ratio, albumin, AST, ALT, and De Ritis ratio values. Postoperative outcomes that were analyzed included hospitalization duration, admission to the intensive care unit, and death within 30 postoperative days. 

### 2.5. Definitions of the De Ritis Ratio and AKI

The De Ritis ratio is calculated by dividing the AST concentration by ALT concentration [6,7]. The preoperative and postoperative De Ritis ratios were calculated within 5 days before and at 1 day after RRP, respectively. 

In this study, we used the Kidney Disease: Improving Global Outcomes criteria for defining postoperative AKI as the serum creatinine concentration increase from the baseline by 0.3 mg/dL up to two postoperative days or by 50% up to seven postoperative days [20,21,22,23]. The AKI stage was defined based on the increase in serum creatinine from the baseline: (stage 1, serum creatinine concentration increase ≥ 0.3 mg/dL or increase ≥ 150–199% compared with the baseline value; stage 2, serum creatinine concentration increase ≥ 200–299% compared with the baseline value; and stage 3, serum creatinine concentration increase ≥ 300% compared with the baseline value, or serum creatinine concentration increase ≥ 4.0 mg/dL, or start of renal replacement therapy) [23]. Because urine output monitoring was not accurate in the general ward, the criterion for urine output was not analyzed in this study. 

### 2.6. Statistical Analysis 

All variables were analyzed for the non-AKI and AKI groups. Continuous variables were analyzed by an unpaired t-test and are shown as the mean ± standard deviation. In other words, categorical variables were analyzed by chi-square or Fisher’s exact tests and are shown as numbers (%). The risk factors for postoperative AKI were identified using univariable and multivariable logistic regression analyses. The most clinically relevant factors related to AKI were analyzed by univariable logistic regression analysis; among these, significant covariates (*p* < 0.05) were selected for multivariable regression analysis using the backward stepwise method. To determine the logistic regression model performance, the C-statistic and the Hosmer–Lemeshow goodness-of-fit statistic were used.

The De Ritis ratio at postoperative 1 day to predict AKI after RRP was analyzed using the area under the receiver operating characteristic (ROC) curve. The optimal cutoff value for the De Ritis ratio associated with postoperative AKI was derived by the maximal sensitivity and specificity. In addition, preoperative serum albumin and eGFR were also analyzed by the ROC curve. Unadjusted and multivariable-adjusted odds ratios (ORs) were used to assess the predictive values of postoperative AKI after dichotomizing by the optimal cutoff value of the De Ritis ratio. Multicollinearities were assessed by examining the variance inflation factor. All statistical analyses performed in this study were conducted using SPSS Statistics for Windows, version 21.0 (IBM Corp., Armonk, NY, USA). The *p*-values < 0.05 were regarded as statistically significant.

## 3. Results

**Figure 1:**
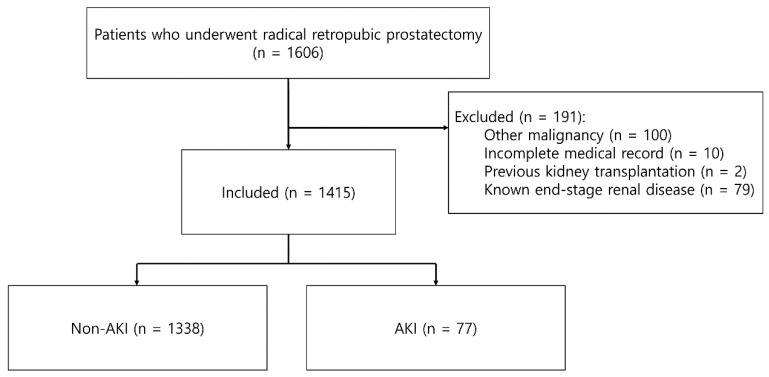
Flow of the study protocol. AKI, acute kidney injury.

A review of medical records led to the identification of 1606 patients who had performed RRP between January 2009 and November 2020. After 191 patients were excluded due to other malignancies (n = 100), incomplete medical records (n = 10), previous kidney transplantation (n = 2), or known end-stage renal disease (n = 79), the final study cohort consisted of 1415 patients (Figure 1). Among these, 77 patients (5.4%) suffered AKI after RRP (stage 1, 45/77; stage 2, 19/77; stage 3, 13/77). Eleven patients (0.7%) were discharged before postoperative 7 days, however, there was no increase in serum creatinine in those patients.

Table 1 shows the 1415 patients’ demographic and preoperative laboratory data; age, ASA physical status, chronic kidney disease, white blood cell, uric acid, and the De Ritis ratio can be seen to have been significantly higher, and lymphocyte count, eGFR, and albumin level can be seen to have been significantly lower among patients with AKI than those without AKI. The intraoperative variables are presented in Table 2. There were no statistically significant differences in the intraoperative variables between the AKI and non-AKI groups. Laboratory data at postoperative day 1 can also be found in Table 2; as can be seen, the hemoglobin level was significantly lower, and the De Ritis ratio was significantly higher among the AKI group in comparison to the non-AKI group.

**Figure 2:**
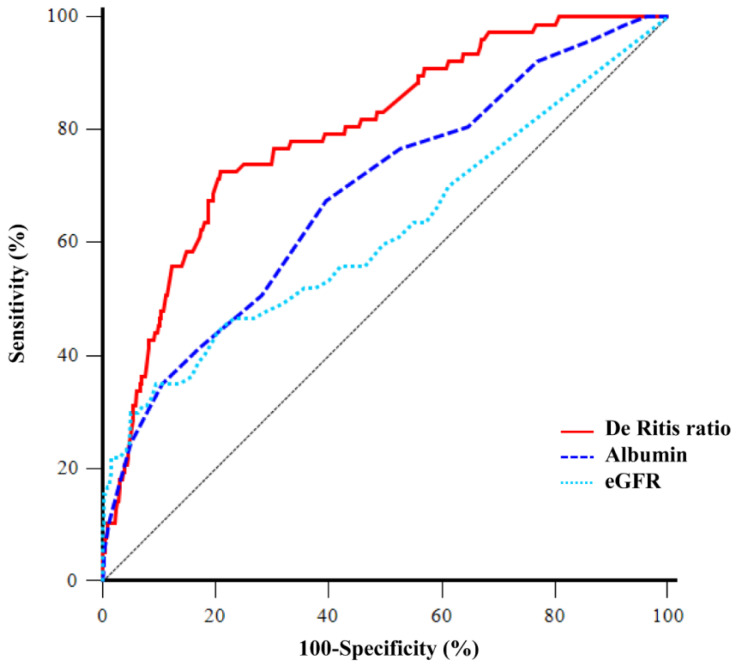
Comparison of ROC curve analyses of the De Ritis ratio at postoperative 1 day, preoperative serum albumin, and preoperative eGFR to predict AKI after RRP. The area under the ROC curve of the De Ritis ratio at postoperative day 1, preoperative serum albumin, and eGFR were 0.801, 0.685, and 0.619, respectively. ROC, receiver operating characteristic; eGFR, estimated glomerular filtration rate; AKI, acute kidney injury; RRP, radical retropubic prostatectomy.

Table 3 shows that the factors related to AKI after RRP are age, white blood cell, eGFR, uric acid, albumin, red blood cell transfusion, and the De Ritis ratio at postoperative day 1 in the univariable logistic regression analysis. Multivariable logistic regression analysis found that eGFR, albumin level, and De Ritis ratio at postoperative 1 day are the significant independent risk factors for postoperative AKI (OR = 0.961, 95% confidence interval [CI] = 0.946–0.977, *p* < 0.001; OR = 0.249, 95% CI = 0.109–0.572, *p* = 0.001; OR = 7.353, 95% CI = 3.967–13.630, *p* < 0.001, respectively). All variance inflation factors were less than 10. The C-statistic and Hosmer–Lemeshow goodness-of-fit statistic were 0.804 and 0.811, respectively. The area under the ROC curve of the De Ritis ratio at postoperative day 1 to predict AKI after RRP was 0.801. The cutoff value of the De Ritis ratio was 1.2 with a sensitivity of 73.8% and a specificity of 79.1% (Figure 2). The area under the ROC curve of preoperative serum albumin to predict AKI after RRP was 0.685. The cutoff value of the serum albumin was 3.9 with a sensitivity of 67.4% and a specificity of 60.3% (Figure 2). The area under the ROC curve of preoperative eGFR to predict AKI after RRP was 0.619. The cutoff value of the eGFR was 63 with a sensitivity of 36.1% and a specificity of 90.4% (Figure 2).

**Figure 3:**
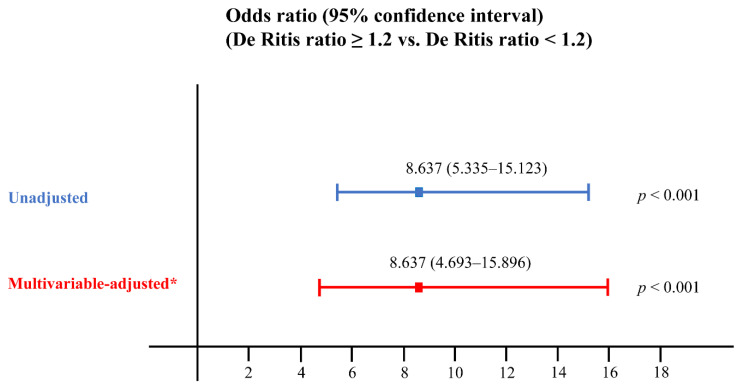
A De Ritis ratio ≥ 1.2 can predict AKI after RRP. The multivariable-adjusted odds ratio was determined using the variables shown in Table 3. The De Ritis ratio was calculated by dividing the AST concentration by the ALT concentration. AKI, acute kidney injury; RRP, radical retropubic prostatectomy; AST, aspartate aminotransferase; ALT, alanine aminotransferase.

Among the study cohort, there were 1020 (72.1%) patients with a De Ritis ratio < 1.2 and 395 (27.9%) patients with a De Ritis ratio ≥ 1.2. Patients with a De Ritis ratio ≥ 1.2 showed a higher association with AKI compared to patients with a De Ritis ratio < 1.2, as revealed by both unadjusted (OR = 8.637, 95% CI = 5.335–15.123, *p* < 0.001) and multivariable-adjusted analyses (OR = 8.637, 95% CI = 4.693–15.896, *p* < 0.001) (Figure 3). 

Table 4 shows the postoperative outcomes. Hospitalization duration was significantly longer among patients with AKI than among those without AKI (11 ± 5 days vs. 10 ± 4 days, *p* = 0.002). 

## 4. Discussion

In this study, among the 1415 patients, 77 (5.4%) had AKI postoperatively. The De Ritis ratio at postoperative day 1, preoperative eGFR, and preoperative albumin level were found to be independent risk factors for AKI in patients who underwent RRP. The optimal cutoff value of the De Ritis ratio as associated with postoperative AKI was found to be 1.2. Patients with a De Ritis ratio ≥ 1.2 showed a significantly higher association with AKI compared to patients with a De Ritis ratio < 1.2. Moreover, hospitalization duration was significantly longer among AKI patients than non-AKI patients. To the best of our knowledge, this study is the first to identify the association between the De Ritis ratio and postoperative AKI.

The incidence of AKI in our study was 5.4%. A retrospective study of 39,369 patients who underwent cardiac and noncardiac surgeries found that 6% of the study population had AKI [24]. Another retrospective study reported that 6.8% of the study patients had AKI resulting in a 13.3% in-hospital mortality rate [5]. However, patients in the intensive care unit developed AKI at a rate of up to 20% [25]. Because our patients in this study were ASA physical status 2 or 3, the incidence of AKI might not have been high. Although the incidence of AKI did not seem high in this study, postoperative AKI is the leading cause of morbidity and mortality. Therefore, these efforts to identify the risk factor of AKI can be clinically meaningful.

Serum AST and ALT levels are biomarkers that are used to determine the liver function or liver disease [6]. The functions of these enzymes are related to carbohydrate and protein metabolism [26]. ALT is found mainly in the liver, whereas AST is widely distributed among several organs, including the liver, brain, kidney, heart, and muscles [26,27]. The ratio of the concentrations of AST to ALT has been a topic of study ever since the introduction of the De Ritis ratio. The De Ritis ratio was initially introduced to evaluate the etiology of acute hepatitis. Additionally, the De Ritis ratio has been widely used to assess the progression of liver dysfunction and to predict liver fibrosis. [6,27]. The De Ritis ratio was reported to be higher in patients with non-metastatic bladder cancer. [28] Furthermore, the De Ritis ratio has also been reported to be a significant prognostic indicator for testicular cancer, renal cell carcinoma, bladder cancer, pancreatic cancer, solid tumor, and oropharyngeal cancer [8,9,10,11,29,30,31]. In patients who have undergone radical cystectomy, an elevated De Ritis ratio is associated with overall survival and disease-specific survival [10,29]. An elevated De Ritis ratio was related to a significantly shorter 10-year cancer-specific survival or higher incidence of invasion in renal cell carcinoma [11,30]. In solid cancers or pancreatic cancers, an increased De Ritis ratio has been significantly related to poor survival [8,31]. Additionally, an increased De Ritis ratio is an independent risk factor for poor survival in oropharyngeal cancer [9], and the De Ritis ratio has been found to be elevated in several clinical circumstances, such as stroke, chronic heart failure, and SARS-CoV-2 [12,13,32]. Therefore, an unexpected elevation of the De Ritis ratio may be associated with poor outcomes in the presence of tissue damage caused by malignancy, stroke, chronic heart failure, or systemic infection. 

In this study, the De Ritis ratio at postoperative day 1 was found to be an independent risk factor for postoperative AKI in patients who had undergone RRP. Major urologic surgery causes a systemic inflammatory reaction due to tissue damage and stress [33]. General anesthesia and postoperative pain may also be related to postoperative inflammation [34]. In addition, malignancies are directly related to chronic inflammation [35]. Specifically, inflammatory pathways and cytokines are important in prostate cancer [36]. Various factors, such as hypoperfusion, inflammation, and neuroendocrine responses, may contribute to the development of postoperative AKI [5]. Above all, inflammation and oxidative stress are important mechanisms affecting renal perfusion, which result in AKI [5]. Decreased antioxidant abilities and increased reactive oxygen species cause progressive renal damage [37]. Reactive oxygen species induce renal vasoconstriction and hypoperfusion, leading to renal injury [38]. Furthermore, the serum AST and ALT are also related to oxidative stress and systemic inflammation and can predict the prognosis of various diseases [39]. Especially, the AST level frequently increases in the presence of tissue hypoxia or ischemia due to hypermetabolism or in the presence of organ damage due to inflammation or apoptosis. However, the ALT level increases in the presence of hepatocyte damage [26]. Accordingly, the AST level may increase in patients with AKI induced by tissue ischemia or inflammation, which in turn may increase the De Ritis ratio. Consequently, an increase in the De Ritis ratio may predict postoperative AKI in patients who have undergone RRP. This was the rationale behind our study.

This study demonstrates that a De Ritis ratio ≥ 1.2 on postoperative day 1 may predict AKI. In clinical circumstances, the optimal cutoff value for the De Ritis ratio varies, ranging from 0.9 to 1.9 [7,9,10,11,32]. In renal cell carcinoma, a De Ritis ratio of 1.42 as a cutoff value can predict the 10-year cancer-specific survival [11]. A De Ritis ratio of 1.3 can predict disease-specific survival in bladder cancer [10]. In our study, we demonstrate that a De Ritis ratio ≥ 1.2 at postoperative day 1 is significantly related to a higher risk of AKI after RRP and that hospitalization duration was significantly longer in patients who suffered postoperative AKI. Therefore, patients who have undergone RRP and subsequently have an elevated postoperative De Ritis ratio should be carefully managed to reduce AKI and the subsequent duration of hospitalization. 

This study also revealed that preoperative eGFR and albumin levels were significantly related to postoperative AKI in patients who have performed RRP. Preexisting renal dysfunction is a predictor for postoperative AKI in both cardiac and noncardiac surgeries [5]. In patients who underwent lung transplantation, preoperative GFR was an important predictor of postoperative renal replacement therapy [40]. Furthermore, our results demonstrated that a decreased preoperative albumin level was significantly related to postoperative AKI. Serum albumin is the major protein, which is essential for plasma oncotic pressure; it also has many other important physiological functions such as free radical scavenging, capillary membrane permeability, and a reservoir for nitric oxide [41]. Several trials have shown an association with decreased preoperative albumin level and AKI [42,43]. A decreased preoperative albumin level may be related to high morbidity, mortality, and AKI [42]. Therefore, clinicians should be cautious to prevent postoperative AKI when preoperative GFR and/or albumin levels are low. 

This study has several limitations. First, because of its retrospective design, we were unable to identify all covariates that may be relevant. Therefore, our study may have been unavoidably influenced by a selection bias. We tried, however, to include almost all covariate factors associated with AKI in patients who underwent RRP. Second, the study was conducted using data from a single hospital. Accordingly, the findings should be interpreted cautiously. Third, we did not evaluate the factors assessed at the same time points such as the intraoperative or postoperative period in the multivariable analysis. However, we have tried to analyze the factors at specific time points that have more predictive ability. Therefore, their influence on our results would be minimal. Lastly, this study was performed on patients who underwent RRP. There is a possibility of different results in patients with different circumstances. Therefore, further studies may be needed for generalizability.

## 5. Conclusions

Postoperative De Ritis ratio ≥ 1.2 was found to be significantly associated with AKI after RRP. Moreover, hospitalization duration was significantly longer in patients with postoperative AKI. Therefore, our results suggest that the De Ritis ratio, which is easily calculated by dividing the AST level by the ALT level, has prognostic value in predicting postoperative AKI after RRP.

## Figures and Tables

**Table 1:** Demographics and preoperative laboratory data.

Variables	All Patients (n = 1415)	Non-AKI (n = 1338)	AKI (n = 77)	*p*-Value
Body mass index, kg/m^2^	24.8 ± 2.7	24.8 ± 2.7	25.1 ± 2.7	0.353
Age, years	67 ± 6	67 ± 6	70 ± 6	<0.001
ASA physical status				<0.001
≤2	379 (86.1%)	351 (88.2%)	28 (66.7%)	
3	61 (13.9%)	47 (11.8%)	14 (33.3%)	
History of abdominal surgery	268 (18.9%)	252 (18.8%)	16 (20.8%)	0.672
Comorbidities				
Diabetes mellitus	252 (17.8%)	234 (17.5%)	18 (23.4%)	0.189
Hypertension	679 (48.0%)	635 (47.5%)	44 (57.1%)	0.098
Coronary artery disease	93 (6.6%)	91 (6.8%)	2 (2.6%)	0.148
Chronic kidney disease	13 (0.9%)	6 (0.4%)	7 (9.1%)	<0.001
Cerebrovascular accident	45 (3.2%)	42 (3.1%)	3 (3.9%)	0.732
COPD	223 (15.8%)	211 (15.8%)	12 (15.6%)	0.965
Medications				
Aspirin	145 (11.0%)	140 (11.1%)	5 (7.6%)	0.367
Clopidogrel	38 (3.0%)	36 (3.0%)	2 (3.4%)	0.695
Calcium channel blocker	310 (23.1%)	295 (23.2%)	15 (22.1%)	0.832
ACEi or ARB	325 (22.9%)	301 (22.5%)	24 (30.0%)	0.121
Beta blocker	89 (6.6%)	83 (6.5%)	6 (8.8%)	0.447
Gleason score, points	6.9 ± 1.0	6.9 ± 1.0	7.0 ± 1.1	0.788
Tumor stage				0.108
1	862 (60.8%)	819 (61.2%)	43 (53.8%)	
2	37 (2.6%)	35 (2.6%)	2 (2.5%)	
3	469 (33.1%)	439 (32.7%)	32 (40.0%)	
4	50 (3.5%)	47 (3.5%)	3 (3.8%)	
Preoperative laboratory values				
PSA, ng/mL	13.5 ± 27.4	13.6 ± 28.0	11.0 ± 10.9	0.076
Hemoglobin, g/dL	14.2 ± 2.9	14.1 ± 1.3	14.7 ± 11.1	0.580
Platelet, 10^3^/μL	223.0 ± 53.4	223.6 ± 52.5	213.7 ± 65.7	0.200
White blood cell, /mm^3^	6.3 ± 1.7	6.3 ± 1.7	6.8 ± 1.7	0.009
Neutrophil, %	56.5 ± 9.3	56.4 ± 9.3	57.8 ± 8.3	0.258
Lymphocyte, %	32.7 ± 8.5	32.9 ± 8.5	30.6 ± 7.7	0.021
Platelet/lymphocyte ratio	121.4 ± 59.6	121.8 ± 60.2	115.2 ± 47.9	0.331
Neutrophil/lymphocyte ratio	2.0 ± 1.2	2.0 ± 1.2	2.1 ± 1.0	0.346
eGFR, mL/min/1.73 m^2^	80 ± 13	81 ± 11	69 ± 23	<0.001
Uric acid, mmol/L	5.5 ± 1.3	5.5 ± 1.3	6.1 ± 1.7	0.005
Albumin, g/dL	4.0 ± 0.5	4.0 ± 0.5	3.8 ± 0.3	<0.001
AST, U/L	24 ± 11	23 ± 8	30 ± 37	0.131
ALT, U/L	23 ± 11	23 ± 11	21 ± 10	0.072
C-reactive protein, mg/L	0.23 ± 0.57	0.24 ± 0.59	0.22 ± 0.27	0.838
De Ritis ratio	1.1 ± 0.5	1.1 ± 0.4	1.5 ± 1.4	0.008

Continuous variables are presented as the mean ± standard deviation, and categorical variables are presented as numbers (%). The eGFR was calculated using the Chronic Kidney Disease Epidemiology Collaboration equation. The De Ritis ratio was calculated by dividing the serum AST concentration by the serum ALT concentration. ACEi, angiotensin-converting enzyme inhibitor; AKI, acute kidney injury; ALT, alanine aminotransferase; ARB, angiotensin II receptor blocker; ASA, American Society of Anesthesiologists; AST, aspartate aminotransferase; COPD, chronic obstructive pulmonary disease; eGFR, estimated glomerular filtration rate; PSA, prostate-specific antigen.

**Table 2:** Intraoperative variables and laboratory data at postoperative 1 day.

Variables	All Patients (n = 1415)	Non-AKI (n = 1338)	AKI (n = 77)	*p*-Value
Intraoperative variables				
Operation duration, minutes	159 ± 39	158 ± 38	163 ± 45	0.317
Crystalloid amount, mL	1644 ± 700	1650 ± 703	1535 ± 642	0.159
6% hydroxyethyl starch amount, mL	259 ± 330	258 ± 331	276 ± 320	0.637
5% albumin administration	56 (4.0%)	53 (4.0%)	3 (3.9%)	>0.999
Red blood cell transfusion	156 (11.0%)	143 (10.7%)	13 (16.9%)	0.091
Extracapsular extension	645 (45.6%)	604 (45.1%)	41 (53.2%)	0.165
Seminal vesical invasion	192 (13.6%)	180 (13.5%)	12 (15.6%)	0.597
Positive surgical margins	523 (37.0%)	494 (36.9%)	29 (37.7%)	0.896
Pelvic lymph node dissection	1259 (89.0%)	1187 (88.7%)	72 (93.5%)	0.192
Laboratory data at postoperative 1 day				
Hemoglobin, g/dL	11.2 ± 1.8	11.2 ± 1.9	10.7 ± 1.3	0.020
Platelet, 10^3^/μL	184.7 ± 58.4	184.8 ± 56.5	183.9 ± 85.6	0.900
Platelet/lymphocyte ratio	210.4 ± 920.4	212.9 ± 940.5	156.5 ± 160.6	0.648
Neutrophil/lymphocyte ratio	6.8 ± 7.4	6.8 ± 7.5	7.2 ± 3.4	0.502
Albumin, g/dL	2.9 ± 0.3	2.9 ± 0.3	2.8 ± 0.4	0.115
AST, U/L	29 ± 77	27 ± 18	61 ± 317	0.330
ALT, U/L	28 ± 30	28 ± 25	27 ± 75	0.910
De Ritis ratio	1.1 ± 0.4	1.1 ± 0.3	1.5 ± 0.7	<0.001

Continuous variables are presented as the mean ± standard deviation, and categorical variables are presented as numbers (%). The De Ritis ratio was calculated by dividing the serum AST concentration by the serum ALT concentration. AKI, acute kidney injury; ALT, alanine aminotransferase; AST, aspartate aminotransferase.

**Table 3:** Logistic regression analyses of factors associated with postoperative AKI.

Variables	Univariable Analysis		Multivariable Analysis	
	OR (95% CI)	*p*-Value	OR (95% CI)	*p*-Value
Body mass index	1.038 (0.956–1.127)	0.370		
Age	1.092 (1.045–1.142)	<0.001		
ASA physical status				
≤2	1.000			
3	1.909 (0.845–4.313)	0.120		
History of abdominal surgery	1.077 (0.613–1.895)	0.796		
Diabetes mellitus	1.470 (0.862–2.506)	0.158		
Hypertension	1.498 (0.949–2.363)	0.083		
Coronary artery disease	0.351 (0.085–1.453)	0.149		
Aspirin	0.654 (0.258–1.655)	0.370		
Clopidogrel	1.145 (0.270–4.888)	0.852		
ACEi or ARB	1.390 (0.842–2.294)	0.197		
Calcium channel blocker	0.982 (0.554–1.742)	0.951		
Beta blocker	1.354 (0.570–3.219)	0.492		
Tumor stage				
1	1.000			
2	1.088 (0.253–4.675))	0.909		
3	1.395 (0.870–2.236)	0.167		
4	1.216 (0.364–4.064)	0.751		
Gleason score	1.030 (0.829–1.280)	0.788		
Preoperative laboratory test				
PSA	0.992 (0.977–1.008)	0.330		
Hemoglobin	1.027 (0.987–1.068)	0.191		
Platelet	0.996 (0.992–1.001)	0.107		
White blood cell	1.154 (1.030–1.293)	0.014		
Platelet/lymphocyte ratio	0.997 (0.992–1.002)	0.287		
Neutrophil/lymphocyte ratio	1.070 (0.936–1.223)	0.323		
eGFR	0.948 (0.935–0.962)	<0.001	0.961 (0.946–0.977)	< 0.001
Uric acid	1.367 (1.153–1.620)	<0.001		
Albumin	0.087 (0.042–0.178)	<0.001	0.249 (0.109–0.572)	0.001
C-reactive protein	0.955 (0.598–1.526)	0.848		
Operation duration	1.002 (0.997–1.008)	0.429		
Crystalloid amount	1.000 (0.999–1.000)	0.110		
6% hydroxyethyl starch	1.000 (0.999–1.001)	0.844		
Red blood cell transfusion	1.928 (1.072–3.471)	0.028		
De Ritis ratio at postoperative 1 day	9.405 (5.504–16.071)	< 0.001	7.353 (3.967–13.630)	<0.001

The De Ritis ratio was calculated by dividing the serum AST concentration by the serum ALT concentration. AKI, acute kidney injury; ACEi, angiotensin-converting enzyme inhibitor; ARB, angiotensin II receptor blocker; ASA, American Society of Anesthesiologists; CI, confidence interval; eGFR, estimated glomerular filtration rate; OR, odds ratio; PSA, prostate-specific antigen; AST, aspartate aminotransferase; ALT, alanine aminotransferase.

**Table 4:** Postoperative outcomes.

Outcomes	All Patients (n = 1415)	Non-AKI (n = 1338)	AKI (n = 77)	*p*-Value
Hospitalization duration, days	10 ± 4	10 ± 4	11 ± 5	0.002
Intensive care unit admission	20 (1.4%)	17 (1.3%)	3 (3.8%)	0.099
Death within 30 days after surgery	2 (0.1%)	1 (0.1%)	1 (1.3%)	0.110

Continuous variables are presented as the mean ± standard deviation, and categorical variables are presented as numbers (%). AKI, acute kidney injury.

## Data Availability

The data used in the present study are available from the corresponding author upon reasonable request.
